# Longitudinal bone loss in patients with stage 3 CKD: a study based on densitometry and HR-pQCT

**DOI:** 10.1007/s11657-026-01728-3

**Published:** 2026-08-19

**Authors:** Kalyanna S. Bezerra de Carvalho, Ana Teresa P. Vieira, Luciene M. dos Reis, Rosa M. R. Pereira, Rosa M. A. Moyses, Rosilene M. Elias

**Affiliations:** 1 LIM 16 do Hospital das Clinicas da Faculdade de Medicina da Universidade de Sao Paulo, Sao Paulo, Brazil; 2 Department of Medicine, Rheumatology Division, Hospital das Clinicas HCFMUSP Faculdade de Medicina, Universidade de Sao Paulo, Sao Paulo, SP Brazil; 3 Universidade Nove de Julho, UNINOVE, São Paulo, Brazil

**Keywords:** Bone mineral density, Chronic kidney disease, Peripheral quantitative computed tomography

## Abstract

***Summary*:**

In a 1-year longitudinal study of 34 patients with stage 3 CKD and stable bone turnover markers there was no deterioration of bony quality. However, HR-pQCT identified impaired bone density in 73.5% of cases at baseline, which was undetected by DXA. Findings suggest early skeletal involvement despite stable renal function.

**Purpose:**

It has been postulated that chronic kidney disease (CKD) is associated with bone loss. However, prospective data are limited, and it remains unclear whether bone loss or microarchitecture chances occurs in the early stages of CKD with stable renal function.

**Methods:**

In this longitudinal cohort study, patients with stage 3 CKD underwent dual-energy X-ray absorptiometry (DXA) and high-resolution peripheral quantitative computed tomography (HR-pQCT), with reassessment after 1 year. Bone biomarkers, including alkaline phosphatase, intact parathyroid hormone (iPTH), intact fibroblast growth factor-23 (iFGF-23), total procollagen type 1 N-terminal propeptide (tP1NP), and β-isomerized C-terminal telopeptide of type I collagen -I (β-CTX-I) were also measured.

**Results:**

Thirty-four patients were included (51 ± 3 years, 44% men). Bone turnover markers remained stable during follow-up. Osteopenia and/or osteoporosis at one or more sites was found in 45.5% of participants. Low bone mineral density (BMD), T-score < -1, was observed in 32.3% (lumbar spine), 29.4% (total hip), 14.7% (1/3 distal radius), and 26.5% (ultradistal radius). HR-pQCT revealed impaired BMD in at least one site (tibia or radius) in 73.5% of cases. Renal function remained stable during follow-up, with no significant changes in bone biomarkers. DXA showed a slight but significant decline in T-scores at the total hip and 1/3 distal radius, whereas HR-pQCT parameters remained unchanged.

**Conclusion:**

In patients with stage 3 CKD and stable renal function, bone microarchitecture identified impaired BMD not fully detected by DXA. Over 1 year, however, only minimal changes in bone parameters were observed.

**Supplementary Information:**

The online version contains supplementary material available at 10.1007/s11657-026-01728-3.

## Introduction

Mineral and bone metabolism disorders in patients with chronic kidney disease (CKD-MBD) enroll a combination of abnormalities of calcium, phosphorus, parathyroid hormone (PTH), vitamin D metabolism, fibroblast growth factor 23 (FGF-23), bone turnover, mineralization, and volume, as well as vascular calcification [1].

Fractures are by far one of the most important CKD-MBD-related adverse outcomes. Fractures are more frequent in patients with CKD than in the general population [2] and are associated with high rates of hospitalization and death [2, 3]. The incidence of fragility fractures increases according to the CKD stage [4]; however, the association with vertebral fractures remains controversial [5]. According to cross-sectional studies based on dual-energy X-ray absorptiometry (DXA), there is a direct association between bone loss at the total hip and the severity of kidney disease [6–8]. Indeed, the risk of hip fracture increases as CKD progresses from stage 3 to 5 [9]. Although fractures are more frequent and more lethal in patients with CKD than in the general population, knowledge about the biochemical and microstructural mechanisms underlying this increased fragility remains limited.

Although some studies have investigated the progression of CKD-MBD-related changes over time, identifying distinct patterns that reflect their interaction, important limitations remain, and longitudinal studies are scarce. Bone microarchitecture data are even more limited. In a previous study, Nickolas et al. [10] evaluated longitudinal bone loss in 53 patients (10 of them on dialysis) with a broad range of kidney function, using DXA, HR-pQCT and biochemical markers. They identified a significant cortical loss in patients with CKD, which was mediated by hyperparathyroidism and elevated turnover. While deterioration of bone quality is an expected finding in late-stage CKD and dialysis, uncertainty remains as to whether microarchitectural alterations already occur in the earlier stages of CKD, when bone turnover markers remain within normal ranges.

In this study, we aimed to address longitudinal changes in bone biomarkers, bone density, and bone microarchitecture in adult patients with stage 3 CKD, in a 1-year follow-up. Our objective was to evaluate what happens to bone health in patients with stable CKD over this period.

## Methods

### Study design and subjects

This is a prospective observational cohort study. Subjects were individuals at least 18 years of age attending the nephrology service at Hospital das Clinicas, Universidade de Sao Paulo. Patients were recruited in the period between September/2016 and February/2019 and followed for 1 year. Eligibility criteria included an estimated glomerular filtration rate (eGFR) indicating stage 3 CKD, according to CKD-EPI equation [11], and the ability to give informed consent. Exclusion criteria were previous use of bisphosphonate, steroid, calcium carbonate, or anticonvulsant drugs, patients with polycystic kidney disease, those receiving hormone therapy, bedridden wheelchair dependent, and obese patients (body mass index—BMI > 35 kg/m^2^). All patients received 25-(OH)D supplementation targeting levels at least 75 nmol/L. None of the patients were receiving hormone therapy.

The Local Research Ethics Board has approved the protocol (CAPpesq #46,565,615.5.0000.0068) and all patients provided informed consent.

### Laboratory measurements

Blood samples were obtained at baseline and 1 year of the protocol. Serum levels of total calcium (tCa) ionized calcium (iCa), phosphate (P), total tissue non-specific alkaline phosphatase (ALP) were determined using routine laboratory techniques. Serum total intact PTH (iPTH) was measured using an electrochemiluminescence assay (E411 Cobas®. Roche Diagnostics, Mannheim, Germany, reference values – RR = 15–65 ng/L) and serum 25-hydroxyvitamin D (25-(OH)D) was measured using a chemiluminescent immunoassay (DiaSorin. Stillwater, MN., USA). Deficiency of vitamin D was defined as 25(OH)D < 75 nmol/L [12]. β-isomerized C-terminal telopeptide of type I collagen (β-CTX-I) and total procollagen type I N-propeptide (tP1NP) serum concentrations were determined by an automated electrochemiluminescence system (E411; Roche Diagnostics. Mannheim. Germany). Intact fibroblast Growth Factor 23 (iFGF-23) was measured using an assay by Immunotopics, San Clemente CA (RR: 11.7—48.6 ng/L). The samples were collected in the morning and were centrifuged and stored at—80 °C.

### Dual-energy X-ray absorptiometry (DXA) and high-resolution peripheral quantitative computed tomography (HR-pQCT) imaging

Areal bone mineral density (BMD) was measured by DXA using Hologic QDR 4500 A densitometry equipment (Discovery model; Hologic Inc., Bedford, MA) at regions representing the central skeleton (lumbar spine and total hip) and Radius (distal 1/3 and ultradistal) based on standard International Society for Clinical Densitometry protocols. T-scores were calculated based on the manufacturer’s reference data, with values for the proximal femur derived from the NHANES III young adult reference population, in accordance with established DXA reporting guidelines. The same experienced technologist performed DXA measurements. The least significant change for BMD measurement was 0.022 g/cm^2^ at the lumbar spine, 0.027 g/cm^2^ at the total hip, 0.014 g/cm^2^ at the total body, and 0.023 g/cm^2^ at the distal 1/3 radius. DXA was performed at baseline and after 1 year of follow-up. The non-dominant forearm and the left tibia of the individuals were immobilized on a carbon fiber shell provided by the manufacturer.

HR-pQCT acquisition (XtremeCT, Scanco Medical AG, Brüttisellen, Switzerland) was performed using the standard scanning protocol (60 kVp, 1.0 mA), and the region of interest was defined using a scout view. The measurements included 110 slices spanning a length of 9.02 mm (voxel size of 82 μm) from the distal end, positioned 9.5 and 22.5 mm proximal to the reference line for the distal radius and tibia, respectively. The HR-pQCT outcome variables used in the analysis were (a) area (cm^2^); volumetric bone mineral density parameters (mg HA/cm^3^): trabecular volumetric bone mineral density (Tb.vBMD) and cortical volumetric bone mineral density (Ct.vBMD); (b) bone structure parameters: bone volume/tissue volume (BV/TV, %), trabecular number (Tb.N, 1/mm), trabecular thickness (Tb.Th, mm), trabecular separation (Tb.Sp, mm); and (c) cortical parameters—cortical thickness (Ct. Th, mm) and cortical porosity (Ct.Po, %) [13]. In terms of precision, HR-pQCT demonstrated coefficients of variation of 0.93–1.41% at the distal radius and 0.25–1.16% at the distal tibia for density measurements, and of 1.49–7.59% at the distal radius and 0.78–6.35% at distal tibia for morphometric measurements. All examinations were conducted by a trained biomedical scientist who also carefully examined each scan for motion artifacts.

### Statistical analysis

Data are presented as mean ± standard deviation or median (25,75) or as frequency, as appropriate. A paired t-test or Wilcoxon was used to compare parameters pre- and post-1 year of follow-up. We used chi-square or Fisher’s exact test to compare nominal variables between these groups. We applied the Spearman coefficient correlation to test the relationship between independent variables. Repeated measures ANOVA with Geisser-Greenhouse correction with post-test when necessary (Dunn’s) was applied to compare laboratory values at baseline, 6 months, and 1 year. Changes in bone measurements obtained from DXA and HR-pQCT were presented as median (25, 75) and calculated as percentage. A p-value < 0.05 was considered statistically significant. Analyses were performed using SPSS version 28.0 (SPSS Inc., Chicago, IL).

## Results

### Baseline cohort characteristics

**Fig. 1:**
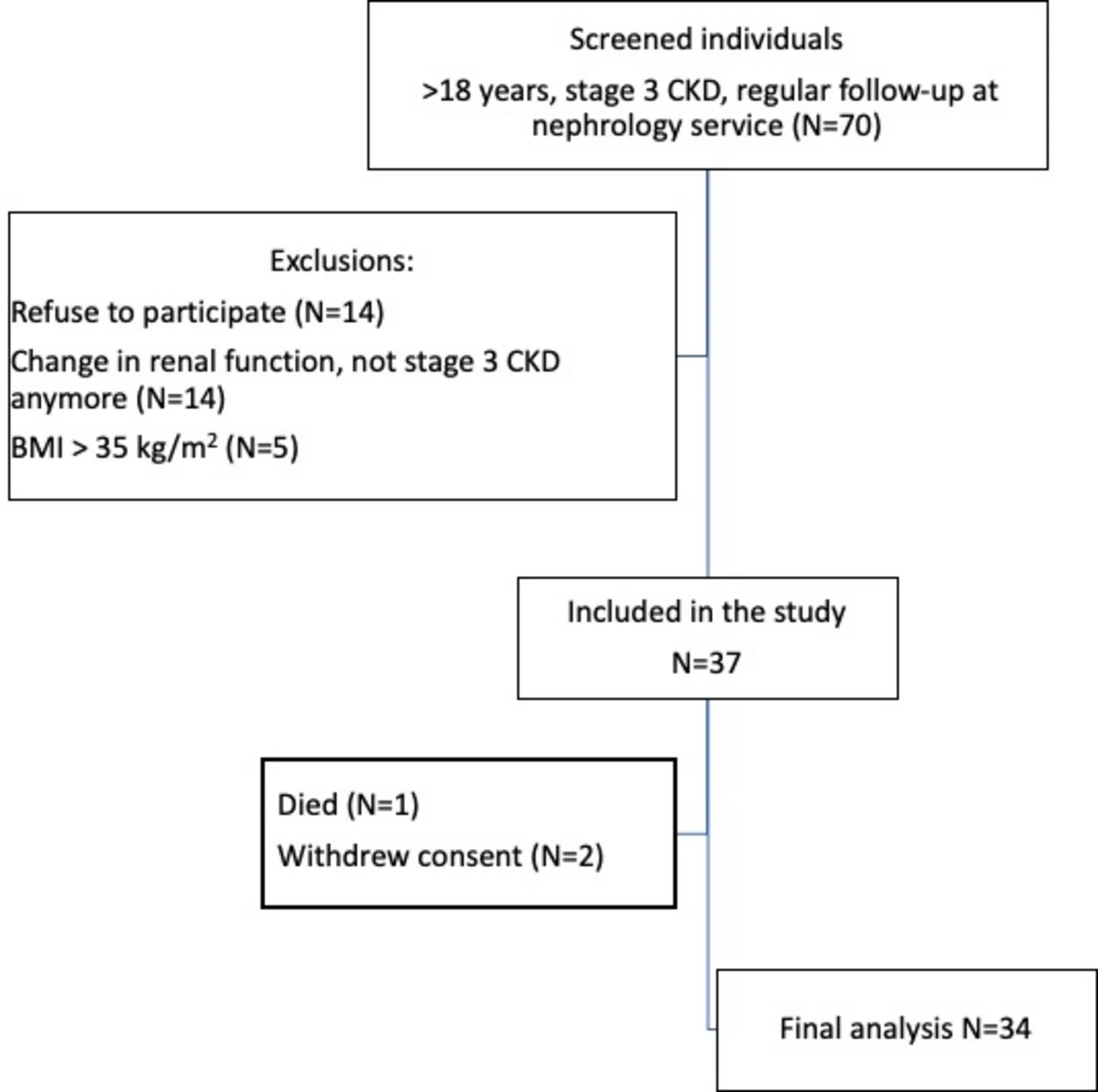
Flow diagram of the study

Out of 70 consecutive screened patients, 14 refused to participate, 14 had an improvement or worsening of renal function in such a way that the CKD stage changed, and 5 had a BMI > 35 kg/m^2^. Therefore, 37 patients were included Before the study initiation, 1 patient died and 2 withdrew their participation (Fig. 1). The final analysis included 34 patients (Table 1). Patients were relatively young, mostly women and non-white. Almost half of the patients were classified as overweight (47%). The etiology of CKD was hypertension in 54.5% of cases, diabetes in 15.2%, glomerulonephritis in 3.0% and other causes in 27.3%. Nine women were considered post-menopausal. Mean levels of iPTH were within the normal range, and only 2 patients had iPTH levels > 100 ng/L. Hypovitaminosis D was found in 17.6% of the patients.

**Table 1:** Characteristics of patients

Parameter	Baseline *N* = 34	1-year follow-up *N* = 34	*p*
Age, years	51 ± 13	-	
Male gender, n (%)	15 (44)	-	
Non-white, n (%)	23 (68)	-	
Weight, kg	74.0 ± 14.0	73.8 ± 14.1	0.817
Body Mass Index, kg/m^2^	27.6 ± 3.6	27.5 ± 3.8	0.538
Furosemide use, n (%)	3 (9)	3 (9)	1
Hydrochlorothiazide use, n (%)	16 (47)	16 (47)	1
Cholecalciferol use, n (%)	10 (29)	10 (29)	1
eGFR, ml/min/1.73m^2^	45.7 ± 8.5	44.6 ± 12.2	0.694
Serum albumin, g/dl	4.5 ± 0.3	4.4 ± 0.4	0.389
Ionized calcium, mg/dl	5.10 ± 0.22	5.22 ± 0.17	0.019
Total calcium, mg/dl	9.7(9.3, 10.0)	9.6 (9.4, 9.9)	0.754
Phosphate, mg/dl	3.4 ± 0.6	3.5 ± 0.5	0.906
ALP, UI/l	72 (59, 89)	83 (67, 102)	0.446
iPTH, ng/L	55 (46, 72)	55 (47, 76)	0.544
25-(OH)D, nmol/L	62.0 ± 19.7	74.5 ± 19.2	0.019
iFGF-23, ng/L	20.2 (15.5, 23.5	23.7 (16.9, 33.0)	0.059
tP1Np, μg/L	53.5 (34.6, 91.2)	56.4 (39.3, 90.2)	0.228
β-CTX-I, ng/ml	0.42 (0.25, 0.70)	0.48 (0.24, 0.77)	0.207
Sodium, mEq/L	141 ± 2.8	141 ± 2.7	0.379
pH	7.34 ± 0.04	7.33 ± 0.05	0.327

*eGFR* estimated glomerular filtration rate, *ALP* alkaline phosphatase, *iPTH* intact parathyroid hormone, *25-(OH)D* 25-hydroxyvitamin D, *iFGF-23* intact fibroblast growth factor 23, *tP1NP* total procollagen type I N-propeptide, *β-CTX-I* β-isomerized C-terminal telopeptide of type I collagen, Collagen type I C-telopeptide Values are expressed as mean ± SD, median (25,75) or percentage

Low BMD (T-score < −1) was found in 32.3%, 29.4%, 14.7%, and 26.5% of patients in the lumbar spine, total hip, 1/3 distal radius, and ultradistal radius, respectively. Osteopenia and/or osteoporosis in at least one bone site was found in 45.5% of the sample. HR-pQCT showed values lower than expected for age and sex [14] in 58.9% and 64.7% of patients in tibia and radius density, respectively. At least one site (tibia or radius) was affected in 73.5% of cases. Of note, trabecular density was altered in 44.1% of tibial and 35.3% of radial sites, whereas cortical density was altered in 26.5% and 50.0% of tibial and radial sites, respectively.

### Follow-up

During the study period, the renal function remained stable, there was no significant change in tCa, P, sodium, alkaline phosphatase, and iPTH, whereas a slight increase in iCa and 25(OH)vitD levels was found (Table 1).

**Fig. 2:**
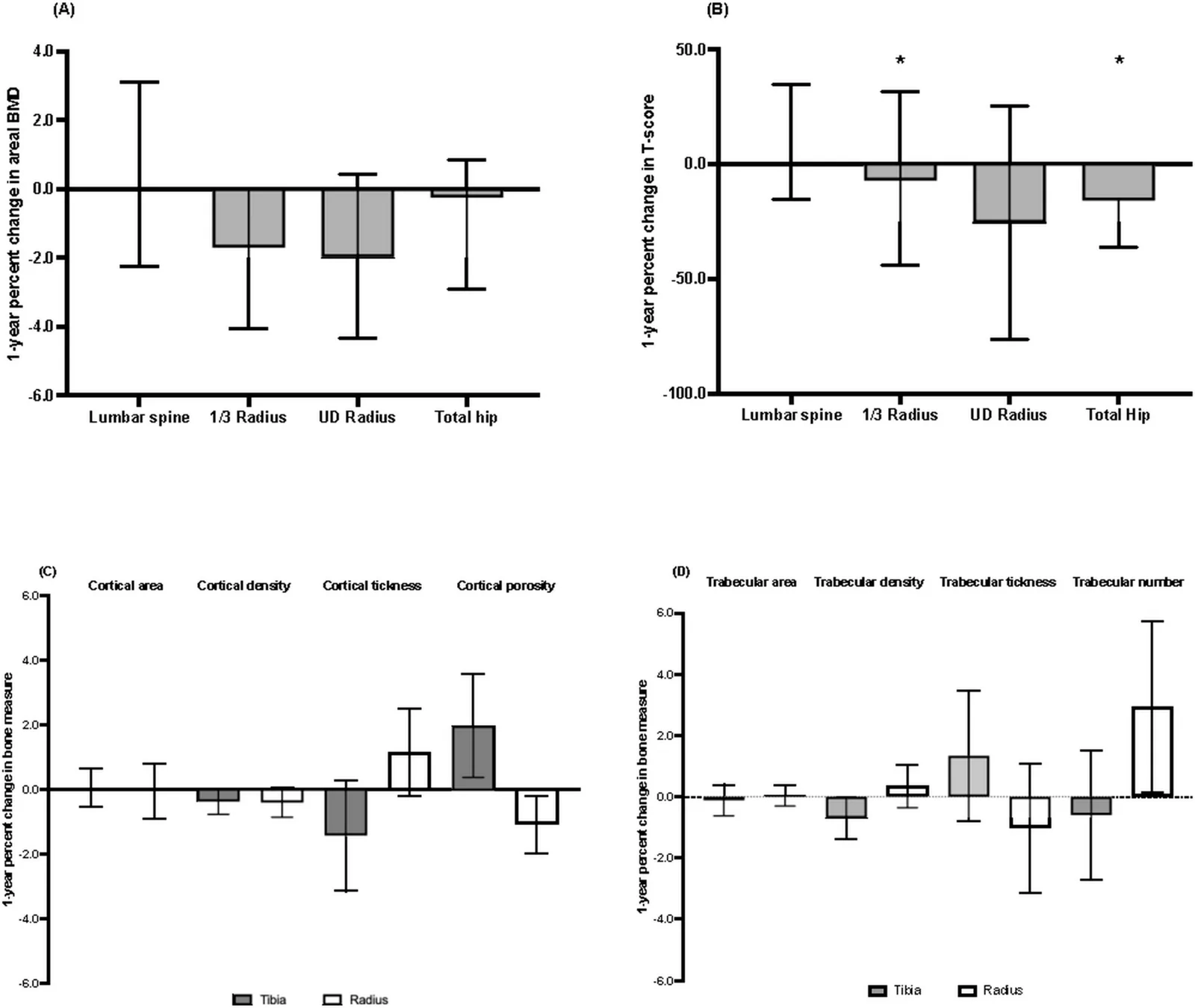
Graphic representation of 1-year percentage change in (**A**) bone mineral density and (**B**) T-scores measured by DXA, and 1-year percentage change in HR-pQCT parameters at the tibia (white bars) and radius (gray bars) in the (**C**) cortical and (**D**) trabecular compartments. Bars represent mean and lines represent the standard error of the mean. BMD, bone mineral density; UD, ultradistal

Supplementary Table 1 shows there was a slight decrease in the total hip and upper 1/3 radius T-score. Neither absolute nor relative change was outside the method precision and, therefore, could not be considered significant. Changes from baseline to 1 year in areal BMD and T-scores are illustrated in Fig. 2A and B. There was a non-significant increase in areal BMD in the lumbar spine, but not in radius or total hip. T-score worsened in total hip and upper 1/3 radius. Figure 2B and C depicted no significant changes in volumetric BMD and bone geometry and microarchitecture obtained by HRpQCT at the tibia (gray bars) and distal radius (white bars).

## Discussion

A major finding of this prospective study was the absence of significant bone loss over a 1-year observation period in patients with stage 3 CKD and stable kidney function, a population with pre-existing bone deficits. While there are some studies on the association between CKD and low bone mass, our study is the first to report on the prospective effect of CKD on bone density and microarchitecture in a 1-year follow-up in this specific population. Neither DXA nor HR-pQCT parameters significantly changed in 1 year. It is noteworthy, however, that HR-pQCT was able to detect reductions in density in a larger proportion of the sample, with greater sensitivity than DXA.

Patients with CKD commonly have bone disease [15]. The areal measurement of bone mineral density (aBMD) by DXA is routinely performed to evaluate bone mass and has been used extensively to evaluate patients with CKD. Indeed, we found low BMD (T-score < −1) in almost half of the study sample (45.5%), in accordance with a previous study from our group, that has shown a prevalence of 32.7% of low BMD (T-score < −1) in a larger sample of 1,172 patients with stages 1–5 CKD [6]. However, DXA does not distinguish between trabecular and cortical bone and may be of limited value in patients with CKD. Compared to DXA, HR-pQCT has the advantage of distinguishing trabecular from cortical BMD and allows measurement of the volumetric density (g/cm^3^) [16]. In fact, a previous cross-sectional study analyzing the association between HR-pQCT and DXA with fractures in individuals with CKD stages 2 to 5, found that the total volumetric BMD at both radius and tibia was most strongly associated with fracture, confirming the higher sensitivity of this method [17]. Although no worsening of BMD was observed over time in our study, baseline HR-pQCT parameters already demonstrated impaired bone density in 73.5% of cases, a proportion not fully identified when relying exclusively on DXA results. This finding, together with the high prevalence of cortical BMD impairment detected by HR-pQCT, highlights the potential underestimation of fracture risk in patients with CKD when only DXA is used.

The finding of a more impressive bone loss at the cortical sites in patients with CKD reinforce the role of iPTH on CKD-associated osteoporosis. In the CRIC cohort, iPTH levels above the reference range were associated with incident fractures [18]. However, most patients included in this study had iPTH values within recommended target. It should be noted that none of the patients have severe hyperparathyroidism, which could have contributed to our results.

Finally, the other risk factor for fracture is aging. In general, the peak bone mass is achieved during youth (by the early 20 s), decreasing with aging, even before sex hormone deficiency [19]. CKD is known to add a bone fracture risk over the effects of aging alone [4, 20]. In the current study, we evaluated relatively young individuals, in their fifties. Also, almost half of them were men, in stage 3 CKD. In this context, we found no significant bone loss. In addition, not only BMD but also bone microarchitecture did not change. Therefore, it may be possible that there is no expressive bone loss in middle-aged patients with stable stage 3 CKD. Our findings are in agreement with a recent publication showing no bone loss in patients with CKD in other sites but radius in a 4.3-year follow-up [21]. The authors postulated that future studies based on HR-pQCT would confirm their results or at least show changes in the microarchitecture not captured by DXA. Our results added some piece of evidence showing there is no change in HR-pQCT parameters in one year of follow-up.

Our findings contrast with those reported by Nickolas et al. [8], who demonstrated significant cortical bone loss at the distal radius in non-dialysis patients with CKD, using HR-pQCT. In the present study, which included only patients with stage 3 CKD and stable renal function throughout the follow-up period, no significant changes were observed in bone mineral density or in cortical and trabecular microarchitecture. In contrast, the above-mentioned cohort comprised older individuals with more heterogeneous CKD stages (2 to 5) and variable renal function, with likely progression in a subset of patients. This clinical distinction may account for the divergent results, as the stability of renal function and bone metabolism markers in our cohort may have contributed to the preservation of cortical bone over one year. In their study, higher iPTH levels in an older population and with possible renal deterioration were associated with accelerated bone loss, suggesting that CKD progression plays a central role in cortical bone deterioration.

The main strengths of this study are the inclusion of a well-characterized population who were monitored by the same research for 1 year, and the assessment based on HR-pQCT which allows evaluation of microarchitecture. The study has also some limitations. Because this is a single-center study, the sample size is small, our population is relatively young, and the period of follow-up is relatively short, the generalizability of our results may be compromised.

In conclusion, patients with stage 3 CKD in their fifties, with stable renal function and no bone turnover marker abnormalities, do not have expressive bone loss during 1 year in any bone site.

The impact of CKD adding a risk factor for reduced BMD might occur later in the course of the disease. To confirm our findings and establish management policies for bone evaluation in the early stages of CKD, further studies with larger patient cohorts and longer follow-up periods are warranted.

## Supplementary Information

Below is the link to the electronic supplementary material.

Supplementary Material 1 (DOCX 18.9 KB)

## Data Availability

The data that support the findings of this study are openly available in [“onehub”] at http://ws.onehub.com/files/pcjqiim5.
