# Supplementary material for: Longitudinal bone loss in patients with stage 3 CKD: a study based on densitometry and HR-pQCT
Source: Arch Osteoporos. 2026 Aug 19;21(1):121. doi: 10.1007/s11657-026-01728-3 (PMC13490007; doi:10.1007/s11657-026-01728-3)
Supplement: Supplementary file 1 [file 11657_2026_1728_MOESM1_ESM.docx]

**Supplementary Table 1.** Absolute and relative changes in bone and mineral parameters in a 1-year follow.

| **Bone densitometry** | **Baseline** | **1-year** | **Absolute change** | **Relative change** |
| --- | --- | --- | --- | --- |
| Lumbar spine BMD, g/cm^2^ | 1.039 ± 0.173 | 1.057 ± 0.194 | 0 (-0.020, 0.031) | 0.02 (-2.25, 3.12) |
| Lumbar spine T-score | -0.564 ± 1.7550 | -0.568 ± 1.563 | 0 (-0.300, 0.200) | 0 (-16.03, 38.09) |
| Total hip BMD, g/cm^2^ | 0.946 ± 0.150 | 0.941 ± 0.168 | -0.002 (-0.024, 0.010) | -0.25 (-2.90, 0.87) |
| Total hip T-score | -0.325 ± 1.258 | -0.457 ± 1.313* | -0.200 (-0.200, 0.100) | -9.95 (-32.35, 6.16) |
| UD radius BMD, g/cm^2^ | 0.467 ± 0.061 | 0.460 ± 0.054 | -0.005 (-0.016, 0.005) | -1.98 (-4.21, 0) |
| UD T-score | -0.436 ± 1.010 | -0.557 ± 1.017 | -0.100 (-0.275, 0.100) | -7.17 (-44.04, 31.66) |
| Upper 1/3 radius BMD, g/cm^2^ | 0.740 ± 0.069 | 0.724 ± 0.064 | -0.014 (-0.028, 0) | -1.70 (-4.07, 0.03) |
| Upper 1/3 radius T-score | -0.425 ± 1.127 | -0.678 ± 1.080* | -0.300 (-0.475, 0.075) | -7.18 (-44.04, 31.66) |
| Total body, BMD g/cm^2^ | 1.111 ± 0.136 | 1.114 ± 0.134 | -0.007 (-0.020, 0.142) | -0.62 (-1.58, 1.16) |
| Total body T-score | -0.700 ± 1.439 | -0.761 ± 1.360 | -0.100 (-0.325, 0.225) | 0 (-19.17, 43.18) |
| **HR-pQCT** |  |  |  |  |
| Tibia Tt.vBMD | 297 ± 59 | 296 ± 59 | -1.450 (-3.475, 0.525) | -0.52 (-1.27, 0.16) |
| Tibia Tb.vBMD | 163 ± 40 | 162 ± 39 | -0.550 (-2.875, 1.450) | -0.33 (-1.80, 1.04) |
| Tibia Ct.vBMD | 888 ± 52 | 885 ± 56 | -2.250 (-13.475, 5.325) | -0.25 (-1.50, 0.58) |
| Tibia Ct.Ar | 122 ± 29 | 122 ± 29 | -0.100 (-1.125, 0.175) | -0.09 (-1.17, 0.15) |
| Tibia Tb.N | 1.84 ± 0.47 | 1.81 ± 0.40 | -0.055 (-0.167, 0.132) | -2.70 (-7.87, 6.31) |
| Tibia Tb.Th | 0.07 ± 0.01 | 0.07 ± 0.01 | -0.005 (-0.001, 0.006) | -0.73 (-7.25, 9.30) |
| Tibia Tb.Sp | 0.50 ± 0.16 | 0.50 ± 0.13 | 0.012 (-0.024, 0.036) | 3.34 (-5.78, 8.67) |
| Tibia Ct.Th | 1.17 ± 0.25 | 1.15 ± 0.27 | 0 (-0.025, 0.010) | 0 (-3.778, 0.872) |
| Tibia BV/TV | 0.14 ± 0.03 | 0.13 ± 0.03 | -0.001 (-0.002, 0.001) | -0.65 (-1.53, 1.40) |
| Radius Tt.vBMD | 325 ± 64 | 323 ± 65 | 0.200 (-6.150, 3.750) | 0.11 (-1.73, 1.32) |
| Radius Tb.vBMD | 179 ± 38 | 178 ± 36 | 1.100 (-3.400, 4.350) | 0.85 (-1.99, 2.28) |
| Radius Ct.vBMD | 861 ± 72 | 856 ± 76 | -2.250 (-13.475, 5.325) | -0.25 (-1.50, 0.58) |
| Radius Ct.Ar | 57 ± 13 | 56 ± 13 | 0.200 (-0.100, 0.650) | 0.35 (-1.58, 1.56) |
| Radius Tb.N | 2.06 ± 0.31 | 2.08 ± 0.28 | -0.010 (-0.130, 0.190) | -0.41 (-7.03, 10.09) |
| Radius Tb.Th | 0.07 ± 0.01 | 0.07 ± 0.01 | 0 (-0.006, 0.004) | 0 (-8.42, 6.44) |
| Radius Tb.Sp | 0.42 ± 0.08 | 0.42 ± 0.07 | 0.001 (-0.041, 0.037) | 0.25 (-9.38, 7.86) |
| Radius Ct.Th | 0.74 ± 0.18 | 0.74 ± 0.18 | 0 (-0.010, 0.015) | 0 (-1.13, 1.65) |
| Radius BV/TV | 0.15 ± 0.03 | 0.15 ± 0.03 | 0.001 (-0.003, 0.003) | 0.93 (-2.26, 2.13) |

BMD, bone mineral density; UD, ultradistal; Tt.vBMD, total volumetric bone mineral density; Tb.vBMD, trabecular volumetric bone mineral density; Ct.vBMD, cortical volumetric bone density; Ct.Ar, cortical area; Tb.N, trabecular number; Tb.Th, trabecular thickness; Tb.Sp, trabecular separation; Ct.Th, cortical thickness; BV/TV bone volume/total volume. Values are expressed as mean ± SD, median (25,75) or percentage.

* p<0.05 vs baseline.
